# Genome-wide view of natural antisense transcripts in *Arabidopsis thaliana*

**DOI:** 10.1093/dnares/dsv008

**Published:** 2015-04-28

**Authors:** Chunhui Yuan, Jingjing Wang, Andrew P. Harrison, Xianwen Meng, Dijun Chen, Ming Chen

**Affiliations:** 1 Department of Bioinformatics, College of Life Sciences, Zhejiang University, Zijingang Campus, Yu Hang Tang Road 866#, Hangzhou 310058, China; 2 James D. Watson Institute of Genome Sciences, Zhejiang University, Hangzhou 310058, China; 3 Department of Mathematical Sciences, University of Essex, Colchester, Essex CO4 3SQ, UK; 4 Department of Molecular Genetics, Leibniz Institute of Plant Genetics and Crop Plant Research Gatersleben (IPK), Gatersleben, Germany

**Keywords:** genome-wide, natural antisense transcript, NAT-siRNA, epigenetic modification, regulation

## Abstract

Natural antisense transcripts (NATs) are endogenous transcripts that can form double-stranded RNA structures. Many protein-coding genes (PCs) and non-protein-coding genes (NPCs) tend to form *cis*-NATs and *trans*-NATs, respectively. In this work, we identified 4,080 *cis*-NATs and 2,491 *trans*-NATs genome-widely in *Arabidopsis.* Of these, 5,385 NAT-siRNAs were detected from the small RNA sequencing data. NAT-siRNAs are typically 21nt, and are processed by Dicer-like 1 (DCL1)/DCL2 and RDR6 and function in epigenetically activated situations, or 24nt, suggesting these are processed by DCL3 and RDR2 and function in environment stress. NAT-siRNAs are significantly derived from PC/PC pairs of *trans*-NATs and NPC/NPC pairs of *cis*-NATs. Furthermore, NAT pair genes typically have similar pattern of epigenetic status. *Cis*-NATs tend to be marked by euchromatic modifications, whereas *trans*-NATs tend to be marked by heterochromatic modifications.

## 1. Introduction

Natural antisense transcripts (NATs) are endogenous transcripts that can form double-stranded RNAs with other transcripts according to the base complementary rule. They can be grouped into two categories: *cis*-NATs and *trans*-NATs, based on whether they work on *cis* or *trans. Cis*-NATs are transcribed from the same genomic locus as their sense transcripts but from the opposite DNA strand, and display perfect sequence complementarity. Whereas, *trans*-NATs are transcribed from separate genomic loci and typically form partial complementarity.^1^ Moreover, *cis*-NATs can be categorized into four subtypes: head-to-head (divergent), tail-to-tail (convergent), fully containing overlap (enclosed) and nearby (nearby head-to-head and nearby tail-to-tail) depending upon their relative orientation and degree of overlap.^2^

Various techniques and methods have been applied to identify NATs from transcriptome data. However, different methods and criteria to scrutinize the data get different results. A review by Werner and Berdal^3^ indicated that ∼22, 15 and 15% genes could form NATs in human, mouse and fly, respectively. But these values are increased to 40.7, 40.9 and 25.6% in a report of Zhang *et al*.^4^ The rice and *Arabidopsis* genome have around 6 and 9% NAT genes.^3^ Wang *et al*.^5^ detected 1,340 potential *cis*-NAT pairs in *Arabidopsis thaliana*, including 957 *cis*-NAT pairs that were certified by massive parallel signature sequencing (MPSS) expression data. However, in this paper, a genome-wide method was used to identify NATs comprehensively, and it was found that 7,962 (23.9%) genes of *A. thaliana* could form NAT pairs, including 4,080 *cis*-NAT pairs and 2,491 *trans*-NAT pairs.

NATs have been proposed to play important roles, especially in regulating the expression of their target genes at several levels, including transcription,^6^ mRNA processing, splicing,^7^ RNA stability,^8^ cellular transport and translational regulation.^9^ Nowadays, more and more studies have focused on the role of plants NATs in post-transcription regulation. For example, Borsani *et al.*^10^ demonstrated that the pair of *cis*-NAT SRO5 and P5CDH has an antagonistic function in the regulation of salt tolerance in *Arabidopsis*. The overlapping region of this NAT pair can produce a 24 nucleotides (nt) siRNA, which could cause post-transcriptional silencing of P5CDH through cleavage. The fragment of cleaved P5CDH could then be used by RNA-dependent RNA polymerase to produce secondary dsRNA, which is subsequently processed by DCL1 to generate the secondary 21nt siRNA, which can also target P5CDH. Endogenous siRNAs from NATs are referred to as ‘natural antisense transcript siRNAs’ (NAT-siRNAs). In plants, the specific siRNAs that derived from the cleavage of the double-stranded RNA (dsRNA) formed by the overlapping region of the *cis*-NATs (termed as ‘*cis-*NAT-siRNAs’) have functional roles in environmental stress responses^10,11^ and in double fertilization.^12^ SiRNAs are more likely to be generated from *cis*-NAT genes than from non-NAT genes.^13^ The density of siRNAs in overlapping regions of *cis*-NAT pairs is greater than in non-overlapping regions.^13^

Chromatin is divided into different functional regions by epigenetic marks, such as DNA methylation and histone modifications. All of these chromatin marks have a substantial effect on chromatin structure and gene function. There are a variety of histone modification types, such as methylation and acetylation. Different histone modifications have distinct distribution and distinct functions. H3K4me3 and H3K36me3 are known as euchromatic marks and are often abundant in highly expressed genes.^14^ H3K4me3 accumulate in the promoters and 5′ genic regions,^14^ while H3K36me3 accumulate across the transcribed region.^15,16^ H3K9me2 and H3K27me3 are major silencing mechanisms in plants.^17^ H3K9me2 is enriched in both the promoter and gene body, which has been found in a limited number of repressed genes in *Arabidopsis*,^18^ while H3K27me3 prefers to mark across transcribed regions which is associated with tissue-specific and developmentally regulated genes.^15,17,18^ Methylation can occur at any cytosine in plants: CG, CHG and CHH (where H = A, C or T). Genes are usually methylated within the promoters (so-called ‘promoter-methylated’), or within the transcribed regions away from the 3′ and 5′ end (so-called ‘body-methylated’).^19^ Promoter-methylation is usually linked to transcriptional silencing, whereas body-methylation prefers exons and likely plays an effect on exon definition during splicing.^20^ Growing evidence shows that NATs are involved in epigenetic regulation, including DNA methylation^21^ and histone modification.^22^ For example, CDKN1A, an antisense transcript of a tumour suppressor gene, can recruit histone-modifying complexes, which induces H3K27me3 to target the promoter region of the sense transcript.^23^ Furthermore, Conley and Jordan^24^ found that *cis*-NATs’ expression are regulated epigenetically in human genome by analysing the chromatin environment of *cis*-NATs’ promoters. In *Arabidopsis*, two transcripts*, COOLAIR* and *COLDAIR*, are related with *FLOWERING LOCUS C* (*FLC*), which encodes a floral repressor in vernalization.^25,26^

NATs are involved in complex regulatory networks. Nowadays, most NATs research focuses on protein-coding genes (PCs), which typically lead to *cis*-NATs. However, long non-coding RNAs (lncRNAs), transposon elements genes (TEs) or pseudogenes should also be taken into consideration. These genome elements have been considered as genomic rubbish, but it is increasingly apparent that they have many roles in organism development. Here, we used a genome-wide method to discover *cis*- and *trans*-NATs of *A. thaliana*, and analysed their multiple aspects, such as expression relationships, production of siRNA and epigenetic patterns.

## 2. Materials and methods

### 2.1. LncRNA prediction and transcriptome analysis

We reanalysed seven RNA-sequencing data sets (total of 18 samples, including four tissues: seedling, leaf, flower and root) for transcriptome reconstruction and lncRNA prediction (Supplementary Table S1). Reads were aligned to the *Arabidopsis* genome (TAIR10) first using Tophat,^27^ and then the resulting alignment files were used to assemble transcripts. Their expression levels (FPKM, fragments per kilobase of transcript per million fragments) were calculated using Cufflinks.^28^ The assembled transcripts were merged together using the Cuffmerge software. The merged assembly transcripts provide a uniform basis for calculating transcript expression in each condition^27^ and the transcript class code. If the class code of transcript is ‘u’, ‘x’ or ‘i’, which means the transcript is intergenic sequence, antisense of known gene or intronic sequence, we took it to be a lncRNA candidate. The CPAT software^29^ was used to assess coding potential of candidate lncRNAs. A candidate was retained if it satisfied: (i) the length should be more than 200 nucleotides; (ii) the maximum ORF length should be no longer than 120 amino acids and (iii) the coding probability cut-off value by CPAT should be <0.365. Furthermore, the transcript which is homologous with any annotated transcript in TAIR10 or with FPKM < 2 in any sample would be removed. Genomic features of these lncRNAs, such as locus and sequences, were obtained using custom Perl scripts.

### 2.2. NATs identification

We assumed that *cis*-NAT pairs are complementary on the same locus and *trans*-NAT pairs are complementary but from different loci, in the same way as in our previous study.^2^ The predicted lncRNAs were complementary to annotated genes in TAIR10 was used as reference of genes. If two transcripts overlap in the genome, they will be selected as putative *cis*-NAT pairs. For *cis*-NATs, they can be grouped into five categories: divergent (head-to-head overlap), convergent (tail-to-tail overlap), containing (full overlap), nearby head-to-head and nearby tail-to-tail according to their relative orientation and degree of overlap. *Trans*-NATs were identified by the following operations. Blast+^30^ was applied to search for potential complementary of NAT transcripts pairs. DINAMelt^31^ was used to verify whether the pairs could melt into RNA-RNA duplexes in the complementary regions. If the pairing region of two transcripts identified by DINAMelt matches with the Blast search, and if any bubble in the predicted pairing region is no longer than 10% of the region, they would be considered as *trans*-NAT pairs.

### 2.3. NAT-siRNAs detection and enrichment in overlapping regions

The enrichment of small RNAs in the overlapping regions of NATs was examined as following: first, for each NAT pair, we have counted the number (*N*_o_) of NAT-siRNAs from our smRNA library mapping to the overlapping region, and the number (*N*_g_) of siRNAs matching with the non-overlapping regions of two genes. If the length of the overlapping region is *L*_o_, and the sum of the lengths of the non-overlapping regions of two genes is *L*_g_, then the density of siRNA loci in the overlapping region is *N*_o_/*L*_o_, and the density of the non-overlapping regions of the two genes is *N*_g_/*L*_g_. For each data set described in this study, we have computed the average densities of siRNA that spawned from NAT genes in the overlapping regions (*A*_o_) and non-overlapping regions (*A*_g_). The ratio *A*_o_/*A*_g_ was considered as the enrichment score (*R*), and a one-tail paired two-sample *t*-test was performed to test the significance of this enrichment.

Forty smRNA-sequencing data sets collected from GEO were used to identify NAT-siRNAs. After removing the reads that match to fRNADB,^32^ such as rRNA, snRNA, snoRNA or miRNA (but preserving tRNA) from the raw data, the filtered sequences were mapped to *Arabidopsis* genome sequences using bowtie.^33^ Then, the smRNA uniquely mapped onto the genome and the length of which is between 18nt and 28nt was selected to constitute our smRNA library. From this library, the smRNA that could be mapped to the overlapping region of a single NAT pair and be discovered in at least three data sets was considered as a NAT-siRNA. We discovered 5,385 NAT-siRNAs, of which 1,548 appeared in three tissues, and 945 and 142 specifically appearing only in seedling and flower, respectively.

### 2.4. Epigenetic analysis of NAT genes and non-NAT genes

In order to explore the difference of histone modification patterns between NAT genes and non-NAT genes, we downloaded the normalized data sets of ChIP-chip data,^34^ including H2Bub, H3K27me3, H3K27me1, H3K4me3, H4K4me2 and H3K36me3.The normalized data were analysed using the MultichipmixHMM method.^35^ Following MultichipmixHMM analysis, neighbouring enriched tiles were joined into domains, with allowing a maximal gap of 200 nucleotides. Only domains of at least 400 nucleotides were considered for further analysis. We acquired genomic locations of these domains using custom Perl scripts.

To investigate the patterns of DNA methylation on NAT genes and non-NAT genes, we obtained ChIP-seq data sets from GEO. Based on the genome-wide DNA methylation sites, we calculated the absolute methylation level^36^ of *cis*-NAT genes, *trans*-NAT genes and non-NAT genes at CG, CHG and CHH sites, respectively.

## 3. Results and discussion

### 3.1. Genome-wide identification of NATs in *A. thaliana*

**Figure 1.**
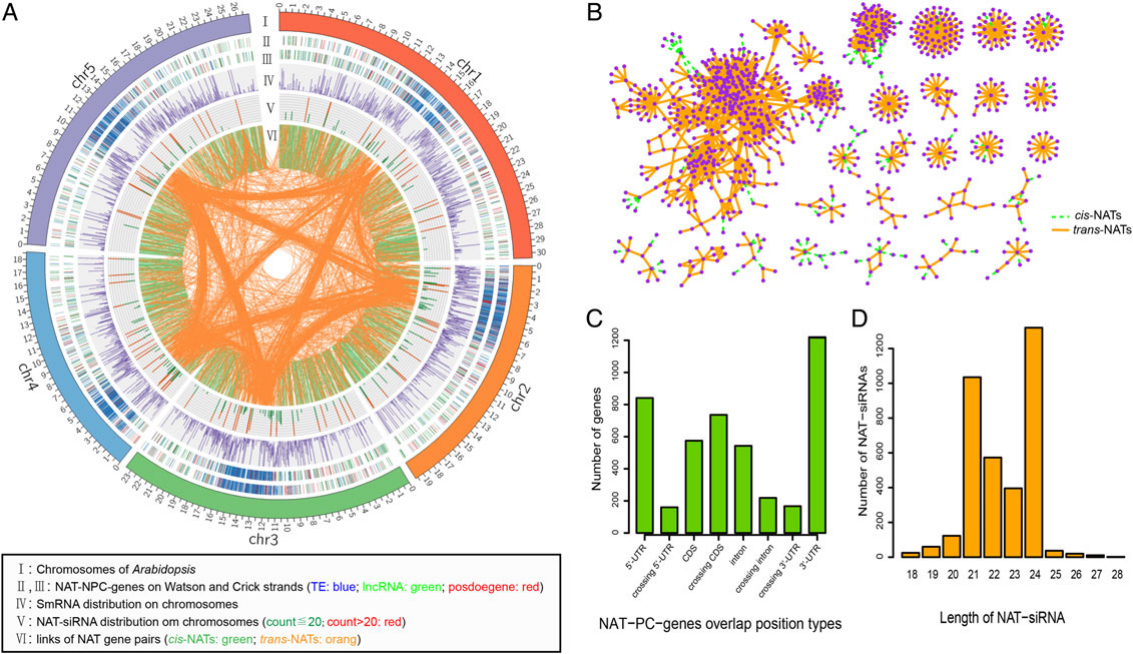
NATs in *Arabidopsis*. (A) NAT genes and NAT-siRNAs in chromosomes. From outermost to innermost, the first track demonstrates *Arabidopsis* chromosomes, the second and third tracks show the NAT genes on Watson and Crick strands, respectively, the fourth and fifth tracks show the distributions of all smRNAs and NAT-siRNAs, innermost links demonstrate the NAT pairs’ loci in chromosomes. (B) The network of NAT pairwises. Node means gene and edge means relationship of NAT pairs. (C) The number of protein-coding NAT genes with different overlapping positions. (D) The number of NAT-siRNAs with different lengths. This figure is available in black and white in print and in colour at *DNA Research* online.

Recent studies have shown that lncRNAs may act as cognate antisense transcripts of other genes^26,37^ which may have roles in regulating gene expression via chromatin-dependent functions. In order to comprehensively identify *Arabidopsis* NATs, we first determined 1,138 lncRNAs, which were complementary to annotated genes in TAIR10. We predicted 6,571 NAT pairs (Table 1), including 4,080 *cis*-NAT pairs and 2,491 *trans*-NAT pairs. Of these 4,080 *cis*-NAT pairs, 1,269 are of types ‘nearby head-to-head’ or ‘nearby tail-to-tail’. Although these are two accepted types of *cis*-NAT, their action mechanism is unknown. We focused our analysis on the other types of *cis*-NATs: convergent; containing and divergent; as well as all *trans*-NATs. Among all transcripts, 7,962 (23.9%) NAT genes from annotated genes (TAIR10) formed NAT pairs and 828 (72.8%) lncRNAs took part in NAT pairs. We discovered that most genes of *trans*-NATs are non-protein-coding genes (NPC) (66.2%), while the genes that are taking part in *cis*-NATs are mostly PCs (79.4%). This indicates that PCs prefer to form *cis-* pair rather than *trans-*, while NPCs have the opposite preference. To demonstrate this feature, the distribution of NAT genes along chromosomes was plotted. From Fig. 1A, we can see that most *trans*-NAT pairs are located in the centriole, where many NPCs are located, thus, showing that NPCs are more likely to be part of *trans*-NATs than of *cis-*NATs. The smRNAs are found across the whole chromosomes with a lower level in the centriole and terminals. Whereas NAT-siRNAs derived from specific regions have a high level at some *trans*-NATs regions. We supposed that NAT-siRNAs were derived from not only *cis*-NAT pairs but also from *trans*-NAT pairs.

**Table 1.** Statistics of genes and candidate NATs in *A. thaliana*^a^

Type	Number
Total genes	33,323
NAT genes	7,322 (22.0%)
*cis*-NAT pairs	3,180
Convergent	1,485
Containing	261
Divergent	192
Nearby	1,242
*trans*-NAT pairs	2,180
Predicted lncRNA	1,138
NAT lncRNA	828 (72.8%)
Total NAT transcripts	8,790
Gene	7,962
lncRNA	828
Total *cis*-NAT pairs	4,080
Convergent	1,616
Containing	811
Divergent	384
Nearby	1,269
Total *trans*-NAT pairs	2,491

^a^In first part only used genes in TAIR10 to predicted NATs. In second part used genes in TAIR10 and lncRNA to predicted NATs.

A NAT network was constructed based on the observed relationships between NAT-genes so as to determine whether any genes are involved in several NAT pairs. Figure 1B shows the subnetworks of the NAT-network, in which the node number is more than 10. From the NAT-network, we found that many transcripts have more than one partners and are involved in several NAT pairs. Gene ontology (GO) enrichment analysis was conducted for each subnetwork, but only the tRNA-hub subnetwork is found to have significant enrichment in GO terms. That may be because most large subnetworks were constructed by NPCs, which are not annotated by GO. It was observed that the biggest subnetwork contains 445 (91.2%) NPCs and most of them could form *trans*-NATs, indicating that NPCs are not only more likely to form *trans*-NAT pairs with other NPC genes but also they have more than one partner. In contrast, PCs always have only one partner to form a *cis*-NAT pair and PCs were hardly ever within a wider subnetwork. The NAT network suggests that the expression of genes may be influenced by other genes in a common network. There are many star-nets of the subnetworks, which suggest that the hub of the net can be matched with many genes to produce NAT pairs. Strikingly, the hubs of stat-nets mostly are tRNA-genes, as previously reported by Zhou in *Oryza sativa*.^38^ Maybe some tRNAs participate in regulation though double-stranded structures; however, this needs more deep studies.

We counted the numbers of overlapping position types in PC NAT-genes to explore which parts of genes are found to overlap. Figure 1C illustrates the positions of PC NAT-genes prefer to form overlaps in the following trend: 3′-UTR > 5′-UTR > across the coding sequence, which is similar to the former observation^13^ that NAT-siRNAs predominantly appear in non-coding regions.

### 3.2. siRNA derived from NAT

To discover if NATs prefer to derive small RNA, we collected 40 small RNA sequencing data sets and obtained a total of more than 100 million raw sequencing reads. After removing the sequencing reads matched to miRNAs, snoRNAs and other small RNAs from fRNADB,^32^ the remaining reads were mapped to the chromosome sequences using bowtie.^33^ Unique sequences with length between 18nt and 28nt were selected for further analysis.

To evaluate whether small RNAs are enriched in the overlapping region rather than non-overlapping region of NATs, we computed the significance of enrichment by *t*-test. The results are shown in Table 2 (all are shown in Supplementary Table S2 sheet of small RNA enrichment). We calculated the enrichment score and found there was no significant siRNA enrichment in the overlapping region. However, it is significant that siRNA enrichment in the overlapping region was larger than in non-overlapping region which is consistent with Zhang's discovery.^13^ Furthermore, the NATs were classified into three subtypes: PC/PC, PC/NPC and NPC/NPC, according to the transcript coding potential, both in *cis*- and *trans*- NATs. To explore if different NATs exhibit inequable raising small RNA, the siRNA density in the overlapping and non-overlapping region of each NAT subtype was calculated. Surprisingly, the enrichment scores of NPC/NPC in *cis*-NATs and PC/PC in *trans*-NATs were large and with significant *P*-values, which means small RNAs are enriched in NPC/NPC pairwise in *cis*-NATs and PC/PC pairwise in *trans*-NATs. So we could conclude that the NPC/NPC pairs in *cis*-NATs and PC/PC pairs in *trans*-NATs prefer to produce small RNAs.

**Table 2.** Enrichment score of small RNAs in the overlapping region of different type NATs

Data set	* *	NATs	*cis*-NATs				*trans*-NATs			
		All NATs	All CIS	PC/PC	PC/NPC	NPC/NPC	All TRANS	PC/PC	PC/NPC	NPC/NPC
Flower										
GSM118372	Score^a^	1.235	1.186	0.589	0.451	17.153	1.276	5.861	2.057	0.666
	*P*-value^b^	0.181	0.347	0.980	0.861	0.026	0.143	0.000	0.096	0.943
GSM154367		1.632	2.744	0.341	0.683	1,742.823	0.845	9.946	16.990	0.014
		0.176	0.081	0.999	0.731	0.028	0.580	0.282	0.034	0.932
GSM280228		2.181	5.477	0.586	5.826	51.199	0.999	3.563	1.314	0.799
		0.056	0.054	0.977	0.138	0.054	0.502	0.002	0.180	0.926
Leaf										
GSM118373		3.788	13.755	0.710	1.184	327.448	0.726	6.640	1.866	0.343
		0.158	0.139	0.812	0.423	0.143	0.713	0.003	0.068	0.874
GSM154370		0.674	3.535	0.816	0.288	1,230.447	0.130	7.651	0.991	0.071
		0.722	0.074	0.583	0.876	0.024	0.937	0.059	0.508	0.944
GSM738727		0.839	1.943	0.569	0.064	172.003	0.276	6.625	1.601	0.104
		0.621	0.213	0.986	0.895	0.030	0.922	0.000	0.058	0.949
Seedling										
GSM118374		1.356	2.600	1.000	0.579	51.480	0.844	11.863	3.809	0.351
		0.191	0.071	0.501	0.792	0.027	0.668	0.002	0.058	0.969
GSM647184		1.034	2.834	0.749	0.388	95.924	0.445	5.021	1.725	0.269
		0.471	0.084	0.880	0.847	0.027	0.897	0.000	0.063	0.937
GSM647187		1.138	2.698	0.729	0.487	90.910	0.581	4.697	2.215	0.319
		0.372	0.078	0.865	0.806	0.025	0.862	0.000	0.064	0.945

^a^The overlapping enrichment score, which captured by the ratio of siRNA densities in overlapping over siRNA densities in non-overlapping (*A*_o_/*A*_g_).

^b^*P*-value for the enrichment score.

To identify the characteristics of NAT-siRNA, 5,385 NAT-siRNAs were predicted from the above smRNA library, of which 1,548 appeared in three tissues simultaneously; with 945 and 142 NAT-siRNAs appearing in seedling and flower, respectively. The observation suggested that NAT-siRNAs are associated with specific tissues. Furthermore, we tested the length distribution of NAT-siRNAs (Fig. 1D), and found that most NAT-siRNAs are mainly 21 and 24 nucleotides.

To investigate which type of NAT prefers to produce NAT-siRNAs, we calculated the number of siRNAs in each of the NAT pair subtype. From PC genes, 1,720 NAT-siRNAs were generated and 3,665 were from NPC genes. Some transcripts might have more than one partner, which means some NAT-siRNAs may be derived from different NAT pair subtypes. We call this type of NAT-siRNA ‘nonunique NAT-siRNA’. Whereas ‘unique NAT-siRNA’ means the NAT-siRNA-derived gene only formed one NAT pairwise. We classified the subtype more clearly into: PC–NPC, PC–PC, NPC–PC and NPC–NPC. The first transcript in the pairwise type is the one that could produce NAT-siRNA, and the second transcript is partner of the first accordingly. The numbers of unique NAT-siRNAs raised from different subtypes in *cis*-NATs are: PC–PC, 366; PC–NPC, 137; NPC–PC, 329 and NPC–NPC, 2,428, meanwhile in *trans*-NATs are: PC–PC, 418; PC–NPC, 421; NPC–PC, 216 and NPC–NPC, 525. This shows that *cis-*NAT-siRNAs mostly originated from NPCs, whereas *trans*NAT-siRNAs mostly originated from PCs. Moreover, most *cis-*NAT-siRNAs from NPCs are 21nt and a large number of *trans*NAT-siRNAs from PCs are 24nt (Supplementary Fig. S1).

### 3.3. The expression of NAT-siRNAs

Diverse Dicer-like enzymes (DCLs) may take part in RNA silencing pathways, such as DCL1, DCL2, DCL3 and DCL4 within plants. DCL1 mainly associates with the production of miRNA and endogenous tasiRNA. DCL2 generates siRNAs from NATs. DCL3 generates siRNA that act as a guide for subsequent chromatin modifications, while DCL4 takes part in inverted-repeat (IR) transgenes silencing.^39,40^ The functions of DCLs are not only specialized and compartmentalized but are also complementary to each other. DCL2 and DCL4 are important in antiviral defence. DCL2 affects alternative processing of siRNA precursors in the absence of DCL4.^41^ The biogenesis of NAT-siRNA is dependent on DCL1, DCL2 and RDR6.^10,11^ Furthermore, Zhang *et al*.^13^ found that the accumulation of NAT-siRNAs required DCL1, DCL3 and RDR2, although they only verified a few NAT-siRNAs, we supposed there should be other feature of NAT-siRNAs’ biogenesis and function.

**Figure 2.**
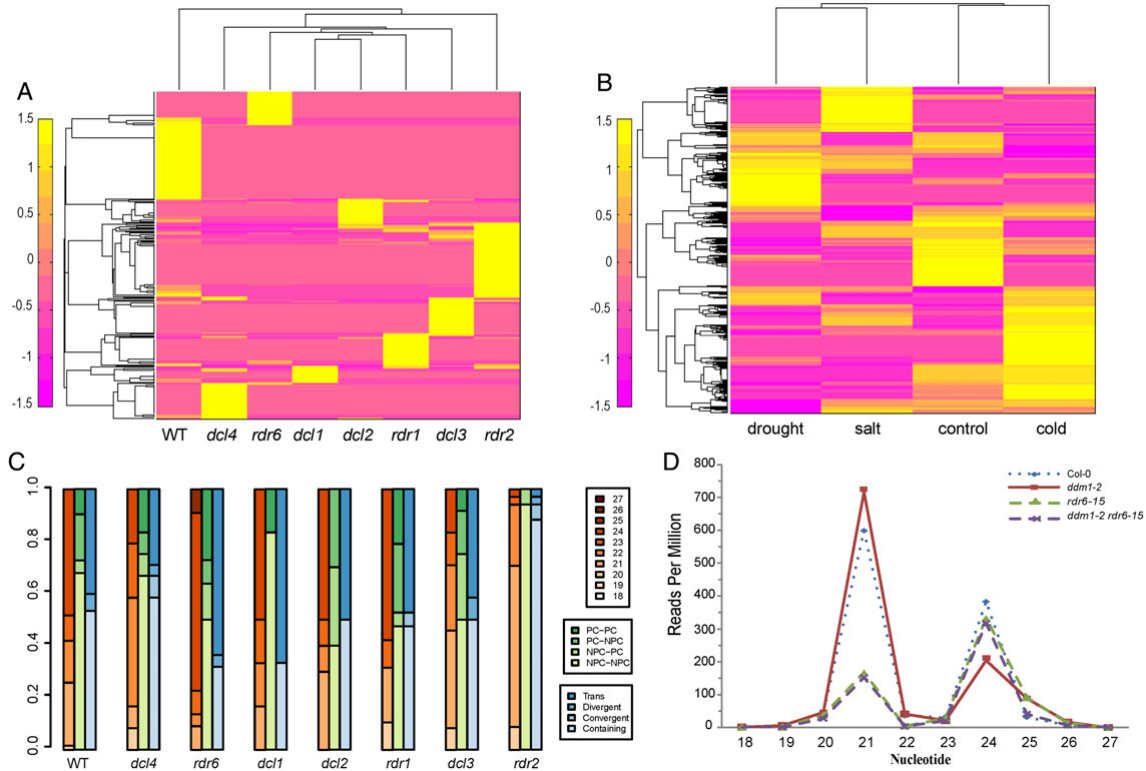
Expression of NAT-siRNAs. (A) The heatmap of NAT-siRNAs’ expression under different mutant samples. (B) The heatmap of NAT-siRNAs’ expression under stress conditions. (C) Diverse types of NAT-siRNAs in different conditions. (D) Reads per million of NAT-siRNAs in mutant samples. This figure is available in black and white in print and in colour at *DNA Research* online.

The profile of NAT-siRNAs suggests that their biogenesis might be through different pathways (Fig. 2A). The sample of the greatest accumulation of NAT-siRNA is *rdr2*, which implies that generation of most NAT-siRNA is RDR2-independent. The length and NAT type in each mutant sample of NAT-siRNAs were further analysed (Fig. 2C). In the *rdr2* sample, most expressed NAT-siRNAs are 21nt and the containing type (*cis* type). However, in the *rdr6* sample, most are 24nt and *trans* type. These results indicated that 21nt NAT-siRNA could be RDR2-independent and 24nt could be RDR6-independent. We concluded that the biogenesis of 21nt NAT-siRNA is by RDR6 and DCL1 or DCL2 pathways and of 24nt NAT-siRNA is by RDR2 and DCL3 pathways.

The expression patterns of 21nt and 24nt *trans*NAT-siRNAs were investigated in the small RNA-sequencing data sets of *ddm1*, *rdr6* and *ddm1 rdr6* (Fig. 2D). We calculated the RPM (reads per million) in each sample. 21nt *trans*NAT-siRNAs accumulated significantly compared with the 24nt *trans*NAT-siRNAs, in *ddm1*. Furthermore, fewer 21nt *trans*NAT-siRNAs were found in *rdr6* and *ddm1 rdr6*. Thus, the generation of 21nt *trans*NAT-siRNAs depends on RDR6 and rises in epigenetically activated *ddm1* mutant. This indicates that 21nt NAT-siRNAs generated from *trans*-NATs are important in repressing the expression of TE, especially in epigenetically activated situations.

The expression analysis of the NAT-siRNAs was studied in four different condition data sets (Fig. 2B). Compared with the expression of NAT-siRNAs in control data set, more specific NAT-siRNAs were expressed under cold environment. Here, also some common NAT-siRNAs appeared in four data sets (Supplementary Fig. S2). By comparing the common NAT-siRNAs and specific NAT-siRNAs, we found that the percentages of *trans*-NATs and 24nt NAT-siRNAs increased in specific conditions, which is presumably because *trans*-NATs and 24nt NAT-siRNAs play a major role in stress responses. These results indicate that NATs, especially *trans*-NATs, have important function in response to environmental pressures.

### 3.4. Histone modifications among NAT genes

**Figure 3.**
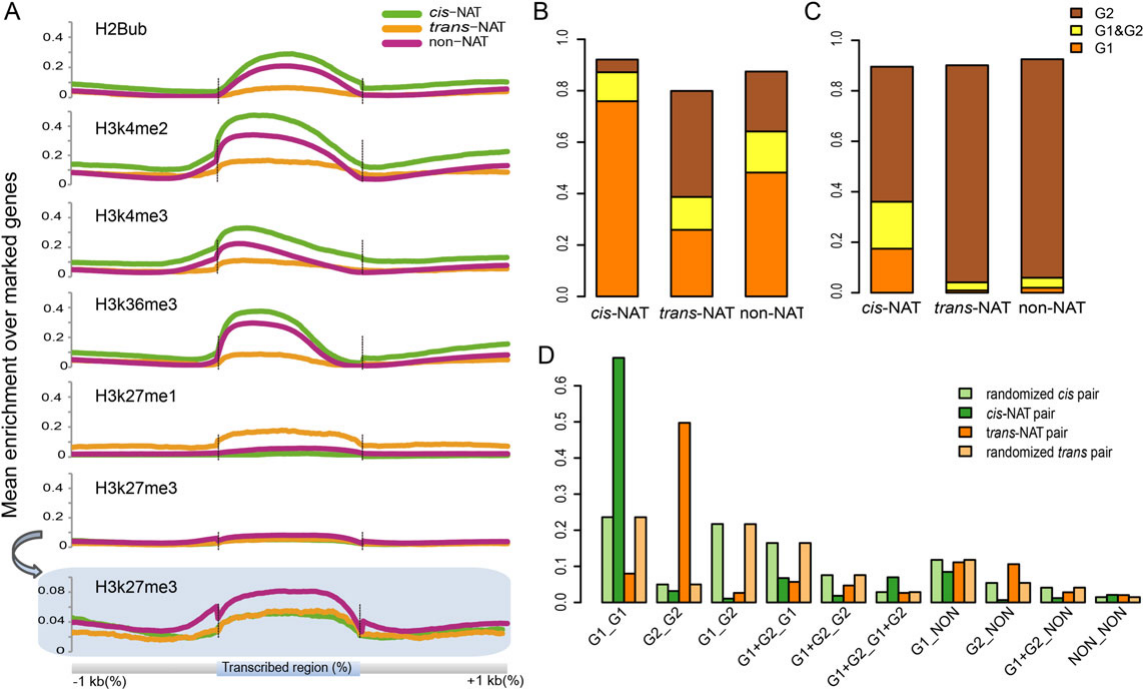
(A) The enrichment degree of histone marks among *cis*-NAT, *trans-*NAT and non-NAT genes. The average enrichment levels are plotted along transcribed region (scaled to accommodate for different gene lengths, bin size of 1%) as well as the upstream (−1kb) and downstream (+1kb) sequences (bin size of 10 bp). (B and C) Histone modification patterns of *cis*-NAT, *trans*-NAT and non-NAT genes. Group1 (G1) means the modifications, H3K4me2, H3K4me3, H3K36me3 and H2Bub, which are euchromatic marks; Group 2 (G2) contains H3K27me1 and H3K27me3, which are heterochromatic marks. G1 and G2 represents genes targeted by marks both G1 and G2. (B) All genes in NAT types and (C) transposon elements only. (D) The divergence histone modifications of two genes in each NAT pair. Different bars shows the percentages of genes of *cis*-NATs, *trans*-NATs, randomized *cis* pairs and randomized *trans* pairs in ten classes of modification patterns (G1_G1, G2_G2, G1_G2, G1+G2_G1, G1+G2_G2, G1+G2_G1+G2, G1+G2_NON, G1_NON, G2_NON and NON_NON). G1, G2 are euchromatic and heterochromatic marks, same as above. NON refers to genes are not marked by above six histone modifications.This figure is available in black and white in print and in colour at *DNA Research* online.

In order to understand the relationship between histone modifications and NAT genes, we have analysed the distributions of six chromatin modifications (H2Bub, H3K27me3, H3K27me1, H3K4me3, H4K4me2 and H3K36me3) along *cis*-NAT genes, *trans*-NAT genes and non-NAT genes. According to the enriched domains definition (Materials and methods section), domains were discovered for each chromatin mark, and then average enrichment levels were calculated within and around genes for all marks. Values are higher within the transcribed region for all the six histone modifications and typically lower in either upstream or downstream regions (Fig. 3A). H3K4me3, H3K4me2 and H3K36me3 accumulated in the transcribed region, and tilted slightly to the 5′-terminal promoter side, especially H3K4me3. In contrast, H2Bub, H3K27me1 and H3K27me3 prefer to mark across transcribed regions, and are higher towards the centre. However, distribution patterns vary for different marks, which are in agreement with previous reports.^15–17,42–44^ Furthermore, these chromatin modifications exhibit preferential association with NATs. Figure 3A shows that active marks are more enriched in *cis*-NAT genes, and illustrates the following enrichment trend: *cis*-NAT > non-NAT > *trans*-NAT genes. However, H3K27me1 is mostly enriched in *trans*-NAT genes, and a bit higher enriched in non-NAT genes than in *cis*-NAT genes. H3K27me3 is mainly enriched in non-NAT genes, with a similar degree of enrichment in *cis*-NAT and *trans*-NAT genes which are smaller than in non-NAT genes.

To investigate whether the chromatin environment of NAT genes differs from non-NAT genes, the epigenetic modifications between them were compared. According to the association with transcriptional activities, six histone modifications could be divided into two groups. Group 1 (G1) includes the following four histone modifications: H3K4me2, H3K4me3, H3K36me3 and H2Bub, which are known as euchromatic marks and often are related with active genes.^15,44,45^ H3K27me1 and H3K27me3 make up the group 2 (G2), which are often known as heterochromatic marks or associated with silenced regions.^34,43^ Our analysis indicates that there are clear differences between the chromatin states for *cis*-NAT, *trans*-NAT and non-NAT genes (Fig. 3B): *cis*-NAT genes tend to be targeted by histone marks from G1, whereas *trans*-NAT genes are mostly marked by histone modifications from G2. One possible explanation for the differential histone modification patterns among these three gene groups is that they have a different percentage of transposon element genes. The majority of TEs are inactive, and there is a steady epigenetic modification state that maintains the TEs silence.^46^ TEs are mostly targeted by regressive marks, rather than active marks. We tested this assumption by calculating the percentage of TEs in three gene groups. As expected, the *trans*-NAT gene group has the largest proportion of TEs (36.5%, 653 of 1,788), and the largest proportion of other NPC genes (29.7%, 531 of 1,788). Although a similar proportion of PCs was observed for *cis*-NAT and non-NAT gene groups (79.4 and 81.2%, respectively), there are more TEs and less lncRNAs in the non-NAT gene group (12.0%, 3,083 of 25,671 and 1.2%, 310 of 25,671, respectively) than in the *cis*-NAT gene group (2.7%, 205 of 7,473 and 11.0%, 821 of 7,473, respectively). Compared with the chromatin status of TEs from *cis*-NAT, *trans*-NAT and non-NAT gene group (Fig. 3C), TEs from the *trans*-NAT and non-NAT gene groups are hardly targeted by marks from G1. But in the *cis*-NAT gene group, more than 35% TEs are marked by histone modifications from G1. All these results indicate that although TEs in the *cis*-NAT gene group are few, they are more likely to be targeted by G1 than those within the *trans*-NAT genes group.

To check the assumption that NAT gene pairs share common histone modification patterns, all NAT pairs were divided into 10 groups according to the modification of each gene in the pairwise (Fig. 3D). To further compare the epigenetic patterns between NAT pairs and randomized pairs in each group, two sets genes were randomly selected as the control sets from non-NAT genes, which have the same gene number with the *cis*-NATs and *trans*-NATs groups. The random selections were performed 10 000 times and the average percentage of each set in each group was calculated. As shown in Fig. 3D, there are different percentages between *cis*-NAT pairs and *trans*-NAT pairs in each group. *Cis*-NAT pairs are mostly marked by group G1_G1, and *trans*-NAT pairs are marked by G2_G2, which indicate the two genes share a common histone modification pattern. Regarding to *cis*-NAT pairs, 67.7% (1,904 of 2,811) are marked by G1_G1, which is greater than the percentage of randomized pairs; however, only 3.2% (89 of 2,811) are marked by G2_G2, which is less than the percentage of randomized pairs. Among the 1,904 *cis*-NAT pairs, marked by activated modification G1_G1, 1,141 (70.6%, 1,141 of 1,616) are convergent pairs, 267 (69.5%, 267 of 384) are divergent pairs and 496 (61.2%, 496 of 811) are containing pairs. This indicates that different types of *cis*-NAT pair show no difference in their epigenetic patterns, and most of them are targeted by active modification marks. In contrast, almost half of (1,239 of 2,491) *trans*-NAT pairs are targeted by G2_G2 and just 8.0% (199 of 2,491) pairs are targeted by G1_G1; 2.6% (66 of 2,491) of *trans*-NAT pairs are marked by oppositely functional epigenetic marks, G1_G2.

### 3.5. DNA methylation among NAT genes

**Figure 4.**
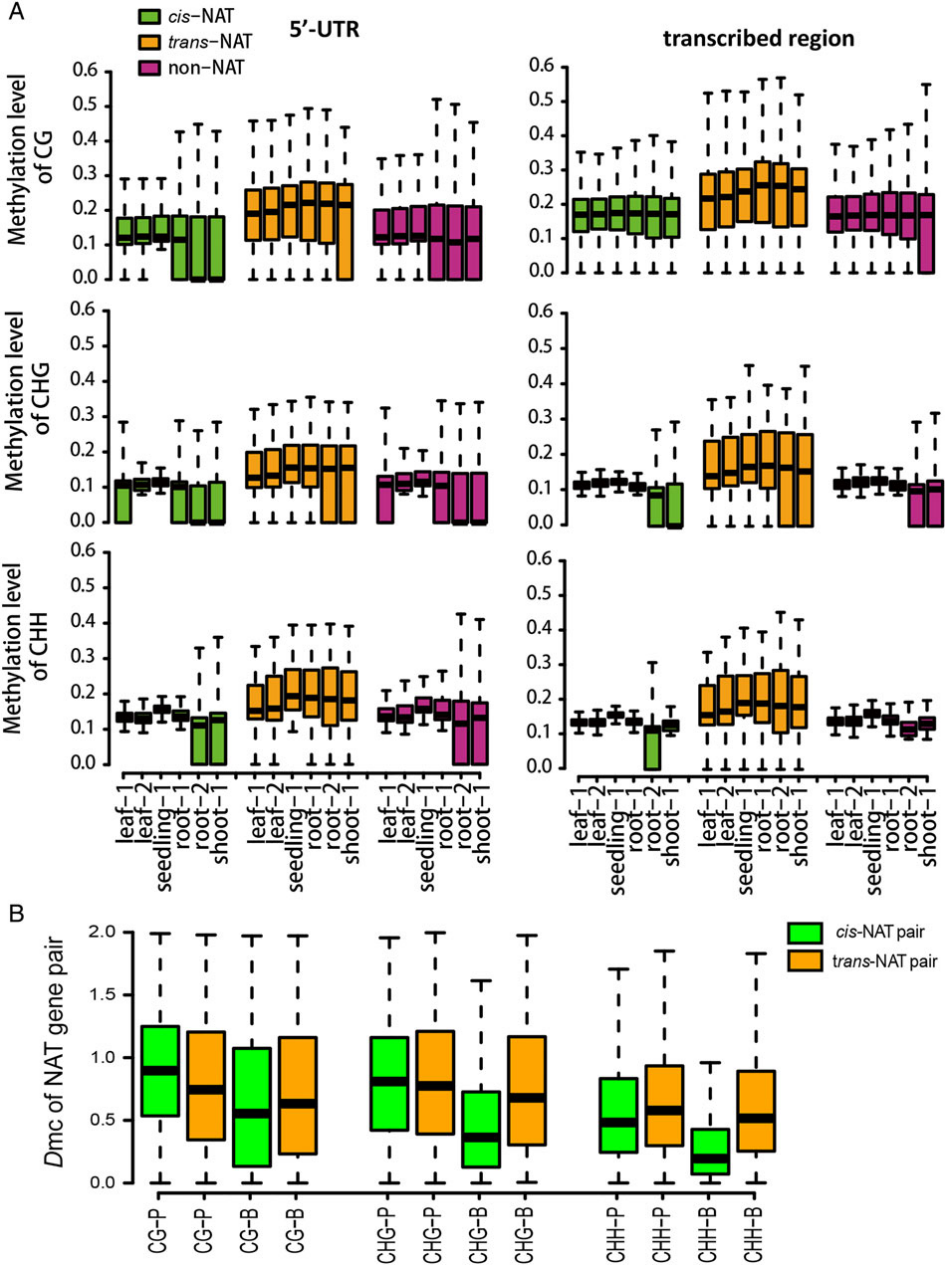
(A) Variation of methylation among *cis*-NATs, *trans*-NATs and non-NATs. The absolutely methylation levels are plotted in 5′-UTR and transcribed region of *cis*-NAT genes, *trans*-NAT genes and non-NAT genes, in six samples respectively. (B) Comparison of DNA methylation divergence between *cis*-NAT pairs and *trans*-NAT pairs at CG, CHG and CHH sites in 5′-UTR and transcribed region. The Y-axis is the average DNA methylation difference of NAT. -P refers to DNA methylated in 5′-UTR, whereas -B refers to DNA methylated in transcribed region. This figure is available in black and white in print and in colour at *DNA Research* online.

Methylation of cytosine in nuclear DNA is a common form of epigenetic modifications that has been reported to exist in mammals and plants. To analyse the relationship of DNA methylation between NAT genes, absolute methylation levels were calculated within and around genes at CG, CHG and CHH sites from six methylation data sets. As shown in Fig. 4A, the DNA methylation level in *trans*-NAT genes is higher than that in *cis*-NAT genes, indicating DNA methylation, as a repression mark, is more enriched in *trans*-NAT genes and less enriched in *cis*-NAT genes. As expected, TEs from the *cis*-NAT gene group had the lowest level of methylation compared with those TEs from the *trans*-NAT and non-NAT gene group (Supplementary Fig. S3).

Previous studies have found some tissue-specific and subfamily-specific hypomethylation of TEs within 928 TE subfamilies in human embryonic and adult tissues by examining genome-wide DNA methylation status. The TEs are located closely to the genes whose functions are important for the relevant tissue type. The hypomethylation of TEs promoted the expression of the surrounding genes, suggesting TEs may be responsible for wiring tissue-specific regulatory networks.^47^ We searched the genes around (2 kb) TEs from the *cis*-NAT gene group and obtained 114 functional annotations from the GO (Supplementary Table S1). According to the function annotation, 12 of the genes adjacent to TEs are involved in response to stresses, such as virus, bacteria and salt. This indicates that those TEs, which are marked by histone modifications from G1 and are less methylated, may play an important role in systemic resistance.

To explore if the two genes from NAT pair have more similar DNA methylation pattern than two randomly selected non-NAT genes, we compared the distance of DNA methylation levels between a NAT pair and a randomized non-NAT pair. We used one minus Pearson's product-moment correlation coefficient, i.e. *D*_mC_ = 1 – *r*, to define the distance of DNA methylation, specifically the distance of DNA methylation at CG, CHG and CHH sites associated in promoter and transcribed regions, respectively. A smaller value of *D*_mC_ indicates a greater similarity of DNA methylation pattern between sense and antisense gene of a NAT gene pair. Randomized pairs were selected from 27 910 non-NAT genes (including lncRNAs) with 10 000 repeats. As expected, we discovered that these six measures show a higher similarity degree of DNA methylation between *cis*-NAT gene pairs than that of randomized non-NAT gene pairs (Wilcoxon's rank-sum test: *P* < 0.05 for all cases). For *trans*-NAT gene pairs, similar results hold (Wilcoxon's rank-sum test: *P* < 0.05 for five cases), except at CHH sites, where *trans*-NAT gene pairs and randomized pairs show no difference in their DNA methylation distance.

We also compared the DNA methylation distance between *cis*-NAT pair genes and *trans*-NAT pair genes. As shown in Fig. 4B, *trans*-NAT pairs have a much greater DNA methylation distance in transcribed regions than *cis*-NAT pairs. Whereas in 5′-promoter regions, *cis*-NAT pairs have a larger methylation distance between sense and antisense gene in CG and CHG except in CHH.

### 3.6. Expression network of NAT genes

**Figure 5.**
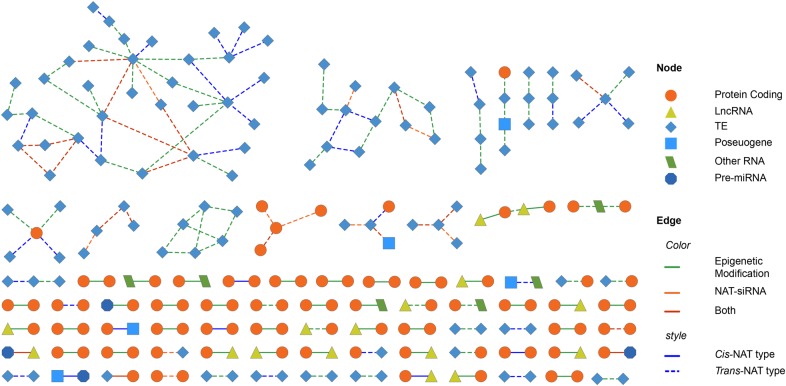
The expression network of NAT-genes. The network was constructed by the NAT pairs with expression correlation |PCC| ≥ 0.8. The solid and dash edge shows *cis-* and *trans-* type of the pair. Both means both of highly similar or distinct epigenetic modification and NAT-siRNA production features happen between this NAT pair. (PCC: Pearson correlation coefficient.) This figure is available in black and white in print and in colour at *DNA Research* online.

Many sense–antisense genes show correlated expression. For example, some *cis*-NATs can regulate the transcript abundance of their complement by triggering the biogenesis of natural antisense siRNA which subsequently guides transcript cleavage.^48^ Chen *et al*.^49^ found that *cis*-NAT gene pairs tend to be coexpressed or inversely expressed more frequently than expected, and they are conserved through evolution. In order to explore comprehensively the correlation between two genes of a *cis*-NAT pair or a *trans*-NAT pair, a NAT expression network was constructed. We used dsRNA-sequencing data sets to determine whether NAT pairs formed double-stranded structures *in vivo*. We counted the average reads that mapped in the overlapping region and also upstream and downstream of this region, then found the dsRNA reads were enriched at overlapping region with significant *P*-values (Supplementary Fig. S4). This indicates that NATs forming double-stranded structure *in vivo* are widespread. One thousand eight hundred and twelve NAT pairs were detected to appear in dsRNA-sequencing data sets. The reads per kilobase per million reads (RPKM) of a gene's expression was counted from the 15 RNA-sequencing data sets. Genes occurring in less than three data sets were removed. Then, the Pearson correlation coefficient (PCC) of the expressions of two genes in a NAT pair was calculated. To show the network clearly, we focused on pairs with correlation |PCC| > 0.8 to construct a network of NAT pairs’ expression (Fig. 5). In this work, the coexpression of NATs is more common than expected. Many TEs are organized into subnetworks and most PC has coexpression relationship with just one partner.

Since many of the NAT genes have an expression correlation and they also have a natural structure relationship, we supposed there might be some regulatory mechanisms shared. To discovery if this is true, we mapped the features of NATs to the expression network. And we focused on the regulation features of NAT-siRNA silencing and epigenetic modifications. We identified coexpressed NAT gene pairs that have the potential to produce NAT-siRNA as well as the coexpressed NAT gene pairs that have a strong epigenetic modification relationship (the absolute value of their PCC of modification pattern similarity is larger than 0.8). Incorporating the feature into the NAT expression network (Fig. 5), we found that most coexpressed NATs have closely related epigenetic modifications. This suggests that some NATs influence each other through their epigenetic modifications and not only through endogenous NAT-siRNAs.

### 3.7. Conclusion

We have made a genome-wide analysis of NATs in *A. thaliana*. To form NAT pairs, 23.9% of genes of *Arabidopsis* in TAIR10 and 72.8% of unannotated lncRNA were discovered, which indicated that most lncRNA prefer to form double-stranded structures. That may be a mechanism by which lncRNAs regulate other transcripts. We predicted 6,571 NAT pairs including 4,080 *cis*-NAT pairs and 2,491 *trans*-NAT pairs. From the predicted NAT pairs, we found not only lncRNAs take part in forming NATs but also TE transcripts. The non-coding transcripts prefer to form *trans*-NAT and PC transcripts prefer to form *cis*-NAT. PC transcripts often only have a single partner to form a pair of NAT; however, NPC have more than one partner and can form several NAT pairs, which leads to a regulatory network. That is a new view about NATs and it indicates different types of genes may have a special function.

One accepted mechanism of NAT regulation is that the antisense transcript could produce siRNA to repress sense transcript expression. By comparing the small RNA density in different regions of the genes, we have shown that small RNA enrichment is significant in the overlapping region of *cis*-NAT pair formed by two non-coding protein transcripts and in the overlapping region of *trans*-NAT pair formed by both coding protein transcripts. Furthermore, these two types of NATs also typically generate NAT-siRNAs. We detected the genome-wide prevalence of NAT-siRNAs and discovered that the lengths of NAT-siRNAs are mainly 21nt and 24nt. Most 21nt NAT-siRNAs are derived from *cis*-NATs, whereas most 24nt NAT-siNRAs are from *trans*-NATs. Based on the NAT-siRNA profiles investigated from smRNA sequencing data sets, 21nt NAT-siRNAs were detected to been processed by DCL1 and/or DCL2, RDR6 and 24nt NAT-siRNAs were found to been processed by DCL3, RDR2. Furthermore, 21nt *trans*NAT-siRNAs have a role in repressing transcripts expression in an epigenetically activated situation. Moreover, 24nt NAT-siRNAs were more likely expressed under stress conditions.

The analysis results of epigenetic modifications show the two genes in a NAT pair have similar histone modifications. *cis*-NAT genes are the most likely to be enriched with active marks, followed by non-NAT and then *trans*-NAT genes. However, heterochromatic marks such as H3K27me1 are enriched in *trans*-NAT genes, even though H3K27me1 is mainly found in non-NAT genes. To explain this observation, we analysed TEs in NATs. Although *cis*-NATs contain less TEs than *trans*-NATs, the percentage of TEs which are targeted by euchromatic histone marks in *cis*-NATs is much more than in *trans-*. We do not know the specific mechanism by which NAT formation influences the modification of TEs or by which specific TEs tends to favour the *cis*-NAT formation, but there are some relations. We also discovered the two genes of NAT pair have similar DNA methylation profiles in the promoter and translated region, with the *cis*-NAT gene pairs more similar than *trans*-NAT gene pairs. Eventually, the methylation levels of CG, CHG and CHH in *trans*-NAT all are higher than in *cis*-NAT.

All the analyses illustrate NAT as a natural formed molecular has important regulation functions both at transcriptional level and at post-transcriptional level. A regulatory network was constructed to illustrate the relationship between NAT genes expression and the potentially regulatory relationship. This indicates the regulation of expression between NATs may be through NAT-siRNA interference and/or epigenetic effects. We hope this study could expand the current views of NATs in plants, and will inspire more research efforts on the detailed regulatory mechanisms of NATs.

## Supplementary data

Supplementary data are available at www.dnaresearch.oxfordjournals.org.

## Funding

This work was supported by the National Natural Sciences Foundation of China (No. 31371328, 31450110068), National Science and Technology Project of China (No. 2009DFA32030); the Fundamental Research Funds for the Central Universities, China; Sino-Germany Cooperation on Agricultural Science and Technology, Germany; and Henry Lester Trust, UK. Funding to pay the Open Access publication charges for this article was provided by the National Natural Sciences Foundation of China.

## Supplementary Material

Supplementary Data
