# Supplementary material for: Genome-wide view of natural antisense transcripts in Arabidopsis thaliana
Source: DNA Res. 2015 Apr 28;22(3):233–43. doi: 10.1093/dnares/dsv008 (PMC4463847; doi:10.1093/dnares/dsv008)
Supplement: Supplementary Data [file supp_22_3_233__index.html]

Genome-wide view of natural antisense transcripts in Arabidopsis thaliana — Supplementary Data 

# Genome-wide view of natural antisense transcripts in *Arabidopsis thaliana*

## Supplementary Data

Supplementary Data

**Files in this Data Supplement:**

- Supplementary Figure 1 - tiff file
- Supplementary Figure 2 - tiff file
- Supplementary Figure 3 - tiff file
- Supplementary Figure 4 - tiff file
- Supplementary Table 1 - xls file
- Supplementary Table 2 - xls file
- Supplementary Table 3 - xls file
