# Supplementary figures and images for: Genome-wide view of natural antisense transcripts in Arabidopsis thaliana
Source: DNA Res. 2015 Apr 28;22(3):233–43. doi: 10.1093/dnares/dsv008 (PMC4463847; doi:10.1093/dnares/dsv008)

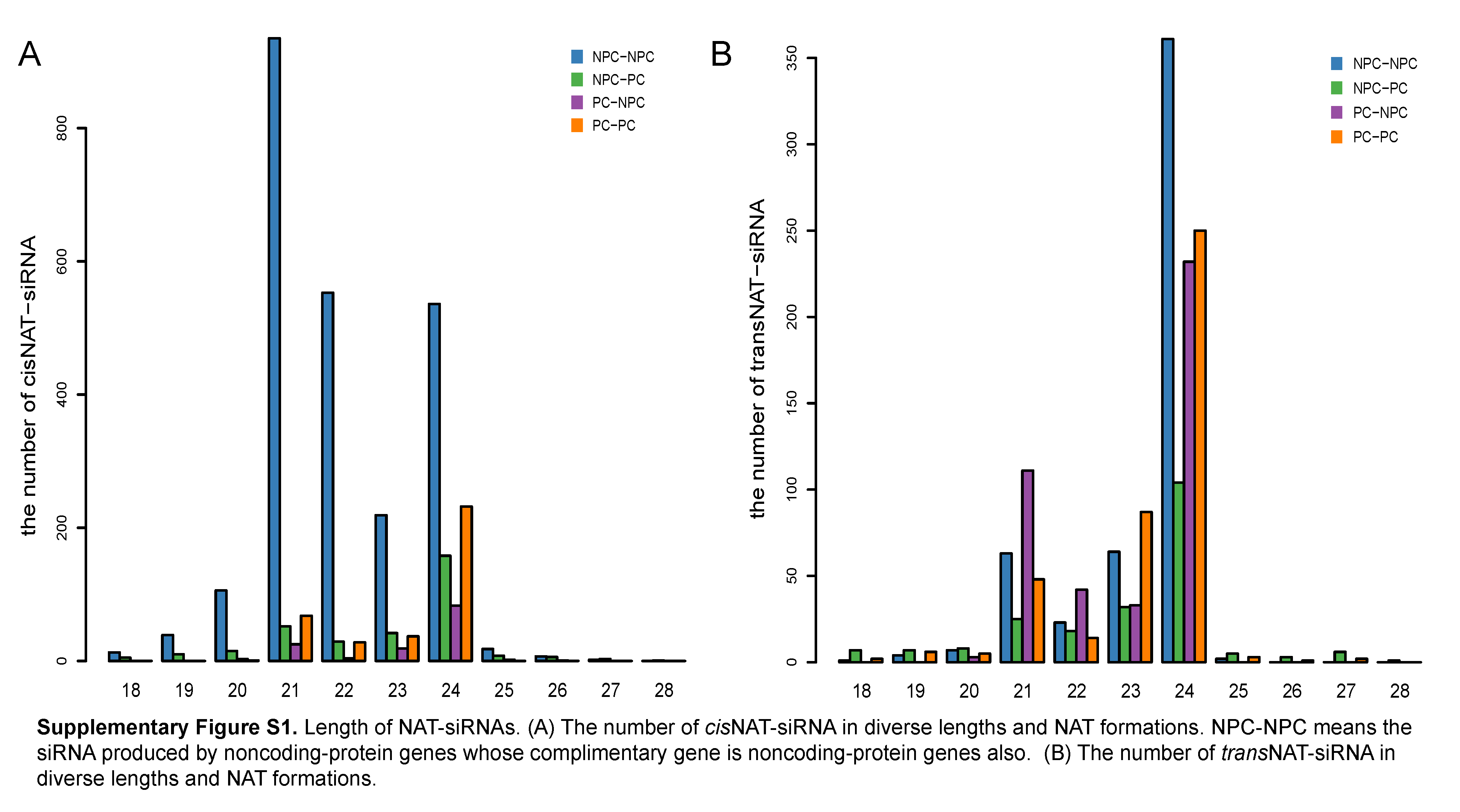

Supplement: Supplementary Data [file supp_dsv008_dsv008supp_fig1.tif]

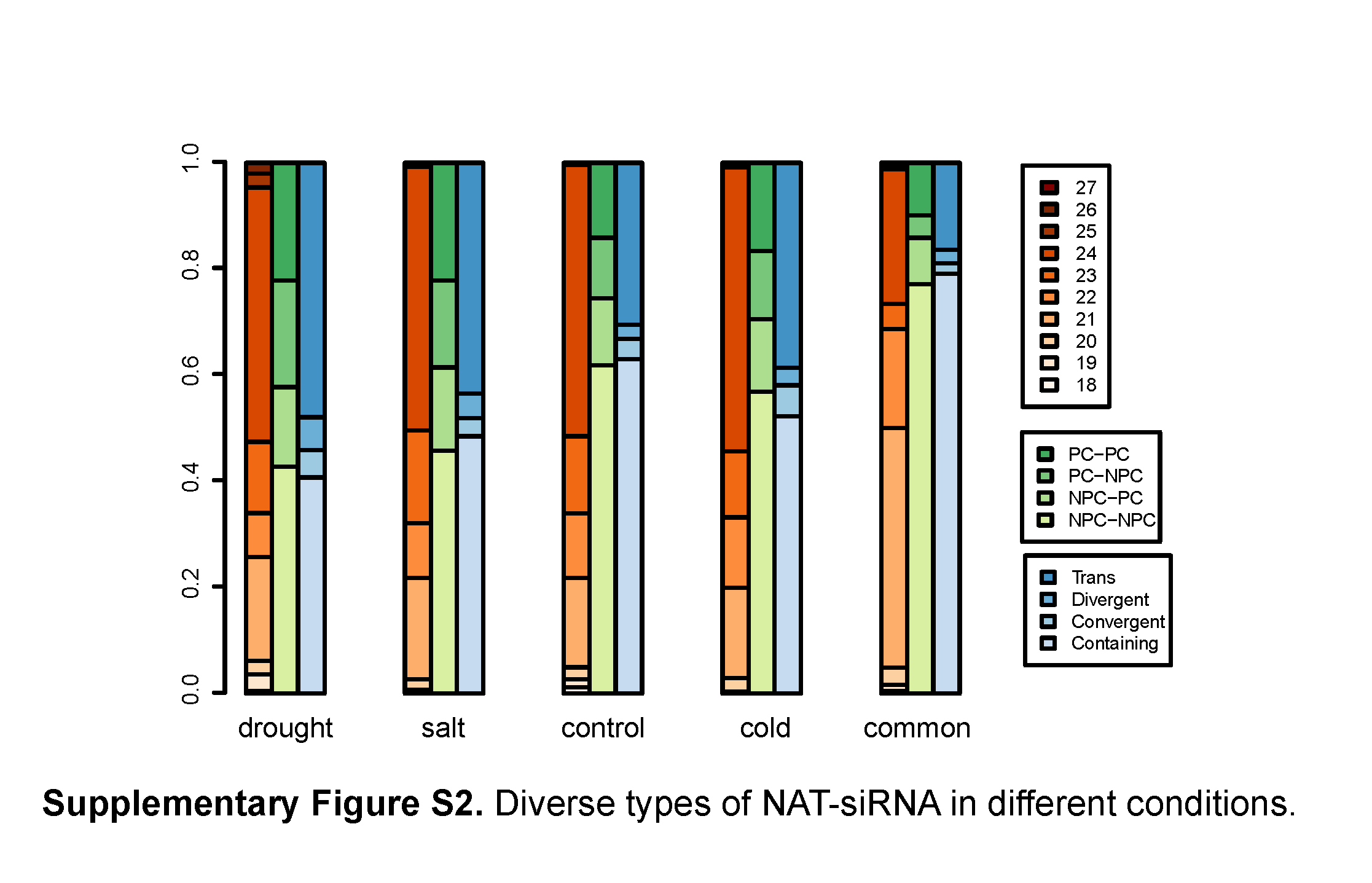

Supplement: Supplementary Data [file supp_dsv008_dsv008supp_fig2.tif]

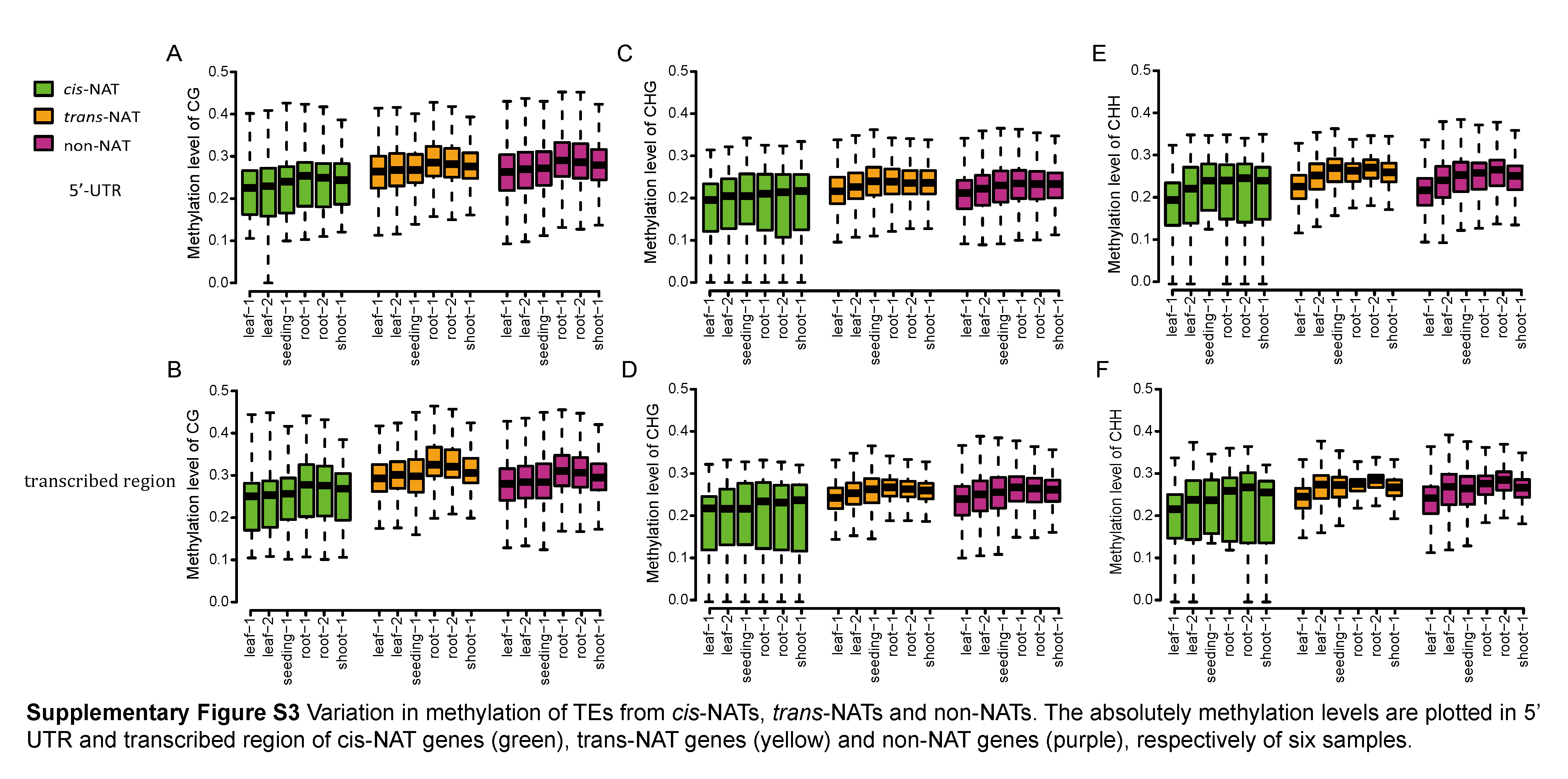

Supplement: Supplementary Data [file supp_dsv008_dsv008supp_fig3.tif]

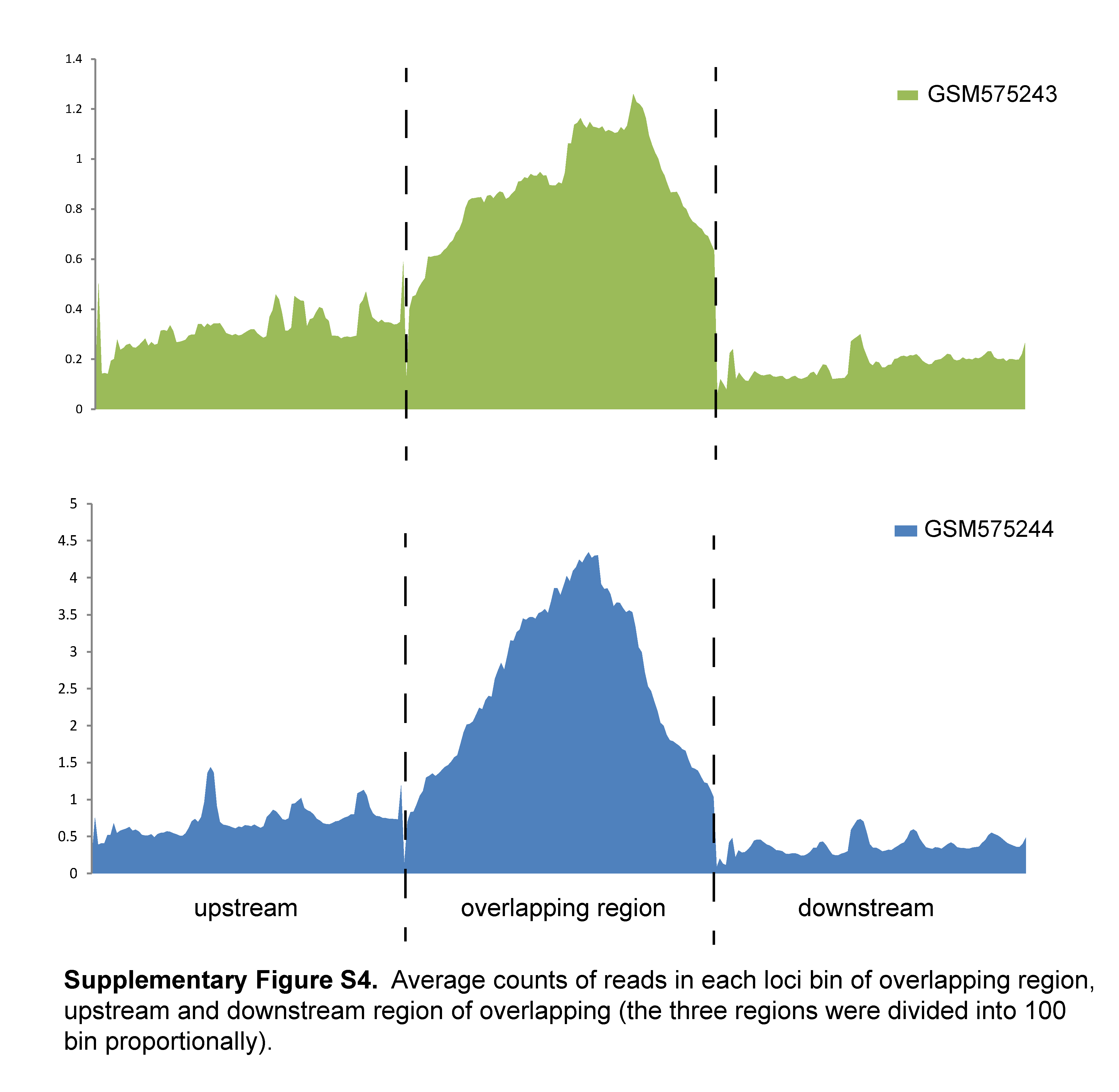

Supplement: Supplementary Data [file supp_dsv008_dsv008supp_fig4.tif]
